# Complications following operative versus nonoperatively managed isolated distal third ulna shaft fractures: a retrospective single-institution cohort study

**DOI:** 10.1007/s00402-026-06509-8

**Published:** 2026-09-23

**Authors:** Kathryn Schultz, Joyce En-Hua Wang, Brock Manley, Kacy Peek

**Affiliations:** 1 Department of Orthopaedic Surgery, University of Virginia Health System, Charlottesville, USA; 2 Department of Orthopaedic Surgery, University of South Florida, Tampa, USA

**Keywords:** Distal ulna, Fracture, Wrist, Forearm, Nonunion

## Abstract

**Purpose:**

This study aimed to compare radiographic and clinical outcomes of operatively versus nonoperatively managed isolated distal third ulna fractures.

**Methods:**

This is a retrospective cohort study. Operatively and nonoperatively managed isolated distal third ulna shaft fractures on forearm radiographs in adult patients with a minimum of six-week follow-up were identified at an academic level 1 trauma center over seven years from 2017 to 2024. Outcomes included rates of radiographic evidence of union, pain scores, nonunion, tendinopathies, removal of hardware, or heterotopic ossification at final follow-up. Outcomes were compared between cases managed operatively and those managed nonoperatively.

**Results:**

A total of 56 isolated distal third ulna shaft fractures were identified with a mean follow-up duration of 30.5 weeks, including 12 (21.4%) managed operatively and 44 (78.6%) managed nonoperatively. The mean age was 49.0 years in the nonoperative group and 38.6 years in the operative group (*p* = 0.115), and the proportions of males were higher in the nonoperative group (61.4% vs. 16.7%, *p* = 0.006). At final follow-up, radiographic evidence of union was present in 77.3% of nonoperative cases and 91.7% of operative cases (*p* = 0.266). The complication rate was lower in the nonoperative group (15.9% vs. 41.7%), although the difference was not statistically significant (*p* = 0.054).

**Conclusions:**

Complication and radiographic union rates were similar in isolated fractures of the distal third ulnar shaft managed operatively and nonoperatively. Both nonoperative and operative management are acceptable treatment options in appropriately selected patients.

## Introduction

Distal ulna fractures have an estimated annual incidence of 74 cases per 100,000 patients and are uncommon as isolated injuries, with over 90% present with concomitant radius fractures [1]. Significant morbidity and functional limitations can result if appropriate healing or alignment is not achieved, resulting in complications such as pain and rotational instability [2,3]. Given the infrequency of these fractures, there is no consensus on optimal management, and comparative evidence regarding management is sparse, with small sample sizes and methodological limitations among studies [4,5]. No absolute indications for surgical fixation of an isolated ulnar shaft fracture exist. Few studies have examined isolated ulna shaft fractures and have not demonstrated superiority of operative versus nonoperative management [6,7].

Distal third ulnar shaft fractures are anatomically and biomechanically distinct from those in the middle third of the shaft due to its proximity to the distal radioulnar joint (DRUJ), the attachment of the central band of the interosseous membrane (IOM), and the triangular fibrocartilage complex (TFCC) [1]. Henry and colleagues compared nonoperative immobilization versus plate fixation of isolated distal and middle third diaphyseal ulnar shaft fractures [8]. This retrospective study demonstrated that in the nonoperative group, distal third ulna fractures are at a lower risk of nonunion when compared to their midshaft counterparts, further suggesting that fractures in the distal third of the ulna present a unique injury and healing profile [8].

Evidence on clinical and radiographic outcomes, as well as guidance on operative versus nonoperative management, is inconclusive for isolated distal one-third ulnar shaft fractures [5]. To better characterize this specific injury pattern and the appropriate management approach, this study aims to compare healing and complication rates in patients with isolated distal third ulnar shaft fractures treated operatively versus non-operatively. It is hypothesized that patients who are surgically treated will have a higher rate of union than those managed nonoperatively at final follow-up.

## Methods

### Study population and design

Institutional review board (IRB) approval was obtained for this study. A retrospective review of the electronic health records at a single academic institution from 2017 to 2024 was performed. Inclusion criteria were skeletally mature patients > 18 years old and the presence of an isolated distal third ulnar shaft fracture, without concomitant ipsilateral radius fracture, on forearm radiographs. Isolated distal third ulnar shaft fractures were defined as fractures within one-third of the full length of the ulnar shaft from the distal tip of the ulna, which includes the metaphysis but excludes the epiphysis.

To create the study groups, all ulna fractures from 2017 to 2024 were first identified using relevant Common Procedural Terminology (CPT) codes (Table 1). Those who underwent open reduction internal fixation (ORIF) were included as the operative group. To supplement cases managed nonoperatively, forearm radiographs obtained in the outpatient hand and upper extremity clinic were reviewed. Patients were excluded if fractures involved the ulnar styloid, distal or proximal radioulnar joints, a coincident middle or proximal ulnar fracture, a radius fracture, or those with less than six weeks of follow-up. Six weeks of follow-up was selected as the minimum follow-up time, as it represents the typical duration of immobilization for nonoperatively managed ulnar shaft fractures in the literature [8,9].

**Table 1:** List of common procedural terminology (CPT) codes included in the search criteria to identify eligible fractures

CPT Code	Description
25,530	closed treatment of ulnar shaft fracture; without manipulation
25,535	closed treatment of ulnar shaft fracture; with manipulation
25,545	open treatment of ulnar shaft fracture, includes internal fixation, when performed
25,560	closed treatment of radial and ulnar shaft fractures; without manipulation
25,565	closed treatment of radial and ulnar shaft fractures; with manipulation
25,574	open treatment of radial and ulnar shaft fractures, with internal fixation, when performed; of radius or ulna
25,575	open treatment of radial and ulnar shaft fractures, with internal fixation, when performed; of radius and ulna

### Data collection

The electronic health records of eligible patients were reviewed to collect physical exam findings, mechanism of injury, return-to-work status, patient demographics, complications, and injury details (displacement, open versus closed injuries, angulation) as documented in clinic and surgical notes. Complication rates were determined for any clinical documentation of complex regional pain syndrome (CRPS), nonunion, removal of hardware (ROH) in the operative group, and development of heterotopic ossification. Subsequent conversion to ORIF of patients in the nonoperative cohort was denoted as nonunion. Pain scores were calculated using the Visual Analog Scale (VAS) obtained at the last follow-up visit. Radiographic union was defined as three of four bridging cortices or osseous consolidation across the fracture site on plain radiographs. Computed tomography (CT) is not systematically utilized at our institution for routine follow-up. Whereas radiographic union was assessed on imaging obtained at the last follow-up visit, nonunion was determined from the entire available medical record through the date of chart review, as patients who required conversion to ORIF have additional encounters and imaging beyond their initial course of nonoperative treatment.

### Statistical analysis

Data were analyzed using an intention-to-treat (ITT) approach, including all participants as originally assigned. Continuous variables were summarized with means and standard deviations (SD), while categorical variables were summarized with counts and percentages. Chi-square and Fisher’s exact tests were used to assess outcome differences between the operative and nonoperative cohorts. Radiographic measurements on injury films were determined to be normally distributed via the Shapiro-Wilk test, and two-sided Student’s T-tests were used for between-group comparisons. Data are represented as mean (Standard Deviation), unless otherwise denoted. All statistical analyses were performed using SPSS (IBM), and p values < 0.05 were considered statistically significant.

## Results

### Demographics

A total of 56 patients with isolated distal third shaft fractures of the ulna with a minimum of six-week follow-up were included, yielding 44 (78.6%) patients in the nonoperative cohort and 12 (21.4%) patients in the operative cohort. The average age was 49.0 (SD: 19.6) years in the nonoperative cohort and 38.6 (SD: 5.6) years in the operative cohort (*p* = 0.115). There was a higher proportion of males in the nonoperative cohort (61.4% vs. 16.7%, *p* = 0.005). The mean duration of follow-up was 30.5 weeks (SD: 62.0; Range: 6–364) for the entire cohort, 28.5 (SD: 61.1; Range: 6–364) weeks for the nonoperative cohort, and 38.1 (SD: 67.3; Range: 6–250) weeks for the operative cohort (*p* = 0.661), and 23 (52.2%) from the nonoperative cohort and 11 (91.7%) completed a minimum of 3-month follow-up. The mean BMI was not significantly different between groups (*p* = 0.831).

### Injury and radiographic characteristics

Table 2 summarizes the demographic and injury characteristics of the study population. The most common mechanism of injury was a motor vehicle collision (*N* = 24, 42.9%), followed by a direct strike to the ulnar aspect of the forearm (*N* = 17, 30.4%), and a ground-level fall (*N* = 9, 16.1%). On initial injury radiographs, patients who were treated operatively had significantly greater displacement on average (6 mm vs. 2.7 mm, *p* < 0.001). There was no significant difference in the degree of fracture angulation (*p* = 0.274) or presence of comminution (*p* = 0.185) between patients treated nonoperatively versus operatively.

**Table 2:** Demographic information, injury mechanisms, and radiographic characteristics by treatment group

Demographics	Nonoperative *N* = 44 (78.6%)	Operative *N* = 12 (21.4%)	*P* Value
Age (mean ± SD)	49.0 ± 19.5	38.6 ± 19.3	0.115
Sex (Male) (%)	27 (61.4%)	2 (16.7%)	0.006
BMI (mean ± SD)	27.6 ± 8.5	27.1 ± 7.0	0.831
Smoking History (%)	16 (36.4%)	2 (16.7%)	0.300
Radiographic characteristics on injury radiographs			
Displacement, mm (mean ± SD)	2.7 ± 2.0	6.0 ± 1.7	< 0.001
Angulation, degrees (mean ± SD)	7.1 ± 5.4	8.7 ± 4.3	0.274
Presence of Comminution (%)	13 (29.5%)	6 (50.0%)	0.185
Injury mechanism			
Motor Vehicle Crash	19 (43.2%)	5 (41.7%)	
Strike to Forearm	14 (31.8%)	3 (25.0%)	
Ground Level Fall	6 (13.6%)	3 (25.0%)	
Crush Injury	3 (6.8%)	1 (8.3%)	
Torsional Force Applied to Forearm	2 (4.5%)	0 (0.0%)	

*SD* standard deviation

### Outcomes and complications

At the most recent follow-up, 34 (77.3%) patients treated nonoperatively and 11 (91.7%) patients treated operatively were deemed healed radiographically, as they had bridging callus on three of four cortices or osseous consolidation across the fracture site at the last follow-up (*p* = 0.266).

**Fig. 1:**
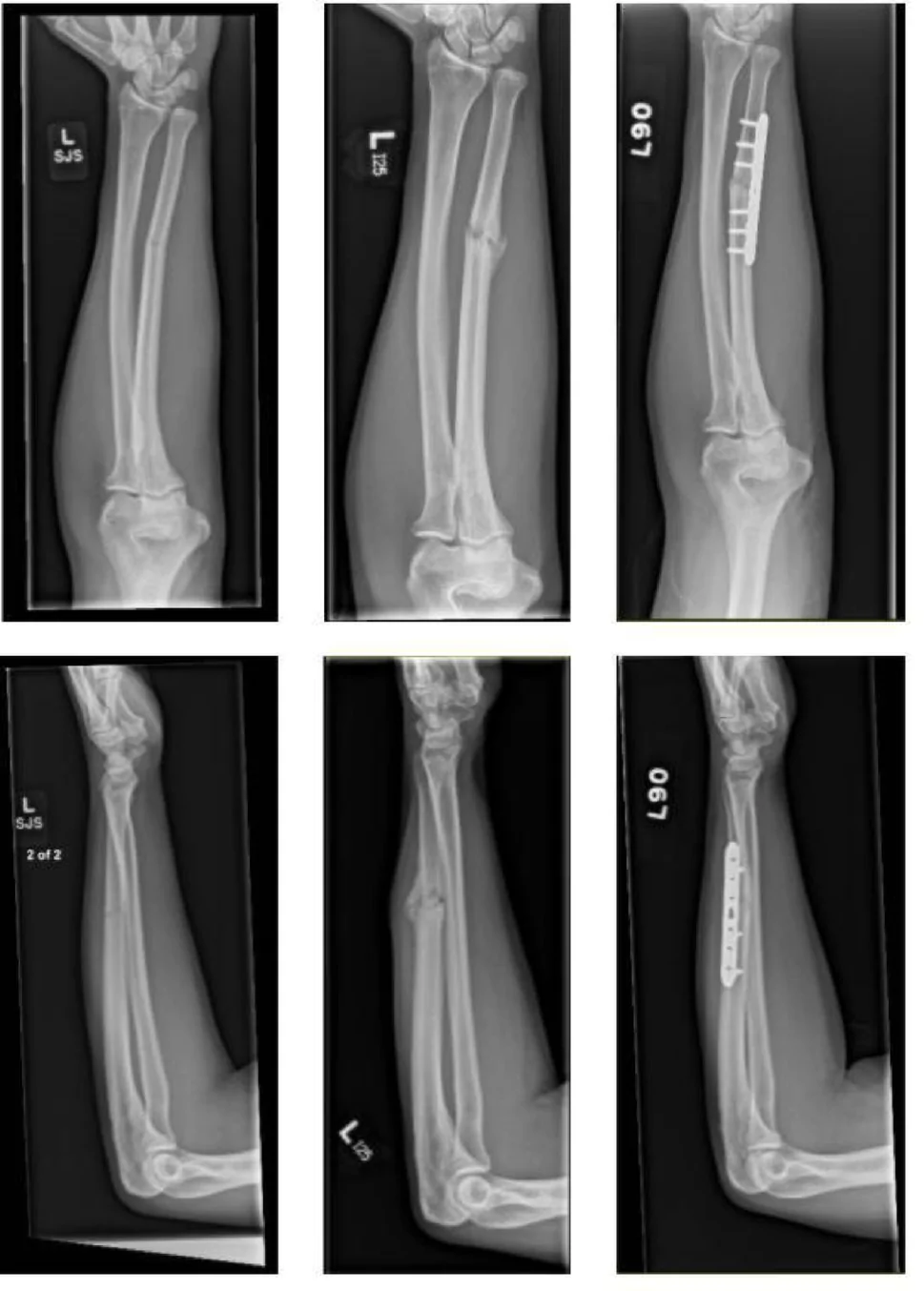
Anteroposterior and lateral forearm radiographs from a patient with a nonoperatively managed distal ulna fracture (left panel) that progressed to nonunion (middle panel) and later healed well following open reduction internal fixation (right panel)

There was no significant difference between the two cohorts in mean VAS pain scores as reported at the last follow-up (*p* = 0.861). Complications listed in Table 3 occurred more frequently in the operative group (*N* = 5, 41.7%) than in the nonoperative group (*N* = 7, 15.9%), although the difference was not statistically significant (*p* = 0.054). By the time of chart review, nonunion was documented in 9.1% (*N* = 4) of patients in the nonoperative group, including two cases that subsequently underwent ORIF (Fig. 1) and none in the operative group. There were two (16.7%) cases of hardware removal in the operative group (Table 3).

**Table 3:** Outcomes at last follow-up and complications following nonoperative vs. operative treatment of distal ulnar fractures

Outcomes	Nonoperative	Operative	*P* Value
Length of follow-up, weeks (mean ± SD)	28.5 ± 61.1	38.1 ± 67.3	0.661
Visual Analog Score (VAS)*	1.84 ± 2.58	2.00 ± 2.86	0.861
Radiographic healing (%)	34 (77.3%)	11 (91.7%)	0.266
Complication rate	7 (15.9%)	5 (41.7%)	0.054
Documented nonunion, including conversion to surgical fixation	4 (9.1%)	0 (0.0%)	
Complex regional pain syndrome	1 (2.3%)	0 (0.0%)	
Tendinopathy	2 (4.5%)	2 (16.7%)	
Removal of hardware	Not applicable	2 (16.7%)	
Heterotopic ossification	0 (0.0%)	1 (8.3%)	

*Data were not available for one subject in the nonoperative group

## Discussion

This study demonstrated that there were no statistically significant differences in nonunion, pain scores, or complication rates in operatively or nonoperatively managed cases. While the optimal treatment selection of isolated distal third ulna shaft fractures remains unclear, nonoperative management can be trialed for stable fractures, though the definition of stability is largely based on data surrounding ulnar shaft fractures at all levels [9]. There are unique anatomical considerations in the distal third of the ulna that create a healing profile different from the middle or proximal portion. Diminishing vascular supply in the distal third of the ulna has been proposed as a deterrent to reliable healing in this portion of the bone [10]. To the authors’ knowledge, this is the first study that compared management approaches in isolated distal third ulnar shaft fractures, as prior studies included fractures in other portions of the diaphysis. In 26 operatively treated and 43 nonoperatively treated distal third ulnar shaft fractures, Henry et al. demonstrated a nearly 100% healing rate at six months in either group. However, nearly a quarter of the nonsurgical patients required conversion to ORIF. In a recent study by Giberson-Chen et al., not all patients who experienced nonunion or malunion from conservative treatment underwent subsequent surgery [11].

Management principles for isolated ulnar shaft fractures are largely based on the stability of the fracture [12,13]. Alter and colleagues defined stability as “those in the middle and distal thirds of the ulna with minimal displacement and angulation” [10]. Zadel and colleagues deemed a fracture unstable when it had “displacement of the proximal segment relative to the distal segment > 50% of the width of the ulnar shaft and/or an angulation > 10◦ between the proximal and distal fragments” [14]. Sauder and Athwal include any involvement of the proximal radial ulnar joint, distal radial ulnar joint, or proximal one-third shaft fracture in their definition of instability [12]. Separately, a 17% failure rate was reported in a recent retrospective study of 154 nonsurgically treated isolated ulnar shaft fractures. An initial fracture gap of ≥ 4 mm or angulation > 10° was identified as a risk factor for failing conservative management [11]. In the present study, fractures that underwent operative management had significantly more displacement on injury films. This finding is consistent with existing literature, where displacement is cited as a predictor of fracture stability [13]. Similarly, in a study by Coulibaly et al., operatively treated isolated ulnar shaft fractures displayed a greater extent of fracture displacement than the nonoperatively treated ones. However, pain, range of motion, and function did not differ between groups [15].

There is mixed comparative evidence on the incidence of nonunion following surgical versus nonsurgical management of isolated ulnar shaft fractures. In the present study, nonunion was not present in the operative cohort, but the nonunion rate in the nonoperative group (9.1%) was higher than rates reported in the literature. In a retrospective review of 46 isolated ulna shaft fractures, the nonunion rate was 25% in the nonsurgical cohort compared to just over 5% in the ORIF cohort [16]. In a prospective randomized trial of 30 isolated ulnar shaft fractures by Hussain et al. (2018), the functional outcomes, elbow and wrist range of motion, did not differ between 14 subjects treated with ORIF or 16 subjects managed with six weeks in a long arm cast at one-year follow-up. Counterintuitively, two ORIF patients and one casting patient developed nonunion in this study. However, this difference in union rate was not statistically significant and likely attributable to the small size of that trial, rather than to a true treatment effect [6]. Among a retrospective review of 254 cases of isolated ulnar shaft fractures, 9.5% (*N* = 21) developed nonunion. The authors reported that surgical fixation did not reduce the incidence of nonunion compared to conservative treatment [17]. When comparing 56 nonsurgically and 39 surgically managed middle and distal third ulnar shaft fractures, Henry et al. found that the nonunion rate was 2.6% (*N* = 1) in surgically managed and 8.9% (*N* = 5) in nonsurgically managed fractures, yet the authors did not determine if the differences between surgical and nontreatment were significant due to inadequate statistical power [8]. While results comparing surgical and nonsurgical management across broader ulnar shaft fracture populations appear conflicting, our study adds to existing literature by specifically characterizing outcomes for isolated distal third ulna fractures.

Various complications can occur after ulnar shaft fractures, including nonunion, malunion, compartment syndrome, and synostosis [17–19]. In this study, the operative cohort demonstrated a higher, although not statistically significant, overall complication rate compared to the nonoperative group. This stands in contrast with the study by Henry et al., where the complication rate was higher among middle and distal third ulnar shaft fractures treated nonoperatively than those treated operatively [8]. Complication rates should be interpreted with caution, as complications of interest vary across studies [6,8,15]. For instance, complications in our operative cohort reflected the additional risks specific to surgical management, such as the potential need for future implant removal [15].

There are several limitations to this study. First, distal third ulnar shaft fractures are not common, and even less common are those that undergo operative management. Additionally, the determination of radiographic healing relied on plain radiographs. Although CT provides a more reliable assessment of bony morphology and union, it was not routinely obtained at follow-up at our institution. As such, subtle differences in early healing may be underappreciated. In addition, the minimum follow-up of six weeks may be insufficient to confirm definitive fracture consolidation. An adequate follow-up of at least three months [20] was available in 60.7% of the sample. Nevertheless, in a post-hoc sensitivity analysis of the 3-month follow-up subgroup, radiographic union occurred in 20 of 23 nonoperative patients (87.0%) and 11 of 11 operative patients (100%). Consistent with the primary analysis, this difference was not statistically significant (Fisher’s exact test, *p* = 0.535). With only 12 operatively treated patients, the study was also underpowered to detect differences in infrequent outcomes such as nonunion based on a post-hoc power analysis, and the higher nonunion rate observed in the nonoperative group (9.1% vs. 0%) did not reach statistical significance. Consequently, our single-institution study may be underpowered to detect clinically meaningful differences between groups. There was also a greater proportion of males in the nonoperative group, and gender-specific differences in healing potential may influence fracture healing [21]. Furthermore, the decision to manage with nonoperative versus operative treatment is based on the surgeon’s preference, which could introduce selection bias. While angulation has been utilized as a marker of fracture instability or outcome [12, 22], the present study did not demonstrate differences in angulation by management approach. However, it should be noted that the average angulation for both operative and nonoperative cohorts was less than 10 degrees, which has been quoted as the acceptable threshold for nonoperative management of ulnar shaft fractures [22]. On the other hand, fractures in the ORIF group were significantly more displaced than those treated nonoperatively. Given the retrospective study design, our findings reflect real-world decision-making around patient and fracture-specific factors, and this study was not powered to derive treatment thresholds. As such, these observations should be regarded as hypothesis-generating rather than as validated indications for surgery. Additionally, heterogeneity within each cohort was present in the immobilization protocols and fixation constructs, which could influence healing and limit the generalizability of study findings. Lastly, the range of motion and functional outcomes were not obtained, as radiographic healing might not correlate with functional improvement. Nevertheless, the present study contributes to the literature by isolating distal third ulnar fractures - a biomechanically and anatomically distinct entity from midshaft ulna fractures. The comparable outcomes between operative and nonoperative cohorts suggest that nonoperative management remains an appropriate treatment in select patients. Larger, prospective studies are warranted to assess long-term outcomes to inform the criteria for operative indications for this fracture pattern.

## Conclusions

In this study, isolated fractures of the distal third of the ulnar diaphysis have low and comparable rates of nonunion whether managed operatively or nonoperatively. Given that greater displacement was observed in operatively treated fractures, treatment selection should be individualized based on patient- and injury-specific factors, and future studies are needed to define operative indications for isolated distal third ulna fractures.

## Data Availability

The datasets generated and/or analysed during the current study are not publicly available due to risks of subject identification, but are available from the corresponding author upon reasonable request.
